# Loop-Mediated Isothermal Amplification (LAMP): Emergence As an Alternative Technology for Herbal Medicine Identification

**DOI:** 10.3389/fpls.2016.01956

**Published:** 2016-12-26

**Authors:** Jing-jian Li, Chao Xiong, Yue Liu, Jun-song Liang, Xing-wen Zhou

**Affiliations:** ^1^College of Forestry and Landscape Architecture, South China Agricultural UniversityGuangzhou, China; ^2^College of Pharmacy, Hubei University of Chinese MedicineWuhan, China; ^3^Sichuan Industrial Institute of Antibiotics, Chengdu UniversityChengdu, China; ^4^College of Biology and Pharmacy, Yulin Normal UniversityYulin, China

**Keywords:** LAMP technology, detection method, herbal medicine, species identification, drug safety

## Abstract

Correct identification of medicinal plant ingredients is essential for their safe use and for the regulation of herbal drug supply chain. Loop-mediated isothermal amplification (LAMP) is a recently developed approach to identify herbal medicine species. This novel molecular biology technique enables timely and accurate testing, especially in settings where infrastructures to support polymerase chain reaction facilities are lacking. Studies that used this method have altered our view on the extent and complexity of herbal medicine identification. In this review, we give an introduction into LAMP analysis, covers the basic principles and important aspects in the development of LAMP analysis method. Then we presented a critical review of the application of LAMP-based methods in detecting and identifying raw medicinal plant materials and their processed products. We also provide a practical standard operating procedure (SOP) for the utilization of the LAMP protocol in herbal authentication, and consider the prospects of LAMP technology in the future developments of herbal medicine identification and the challenges associated with its application.

## Introduction

**FIGURE 1:**
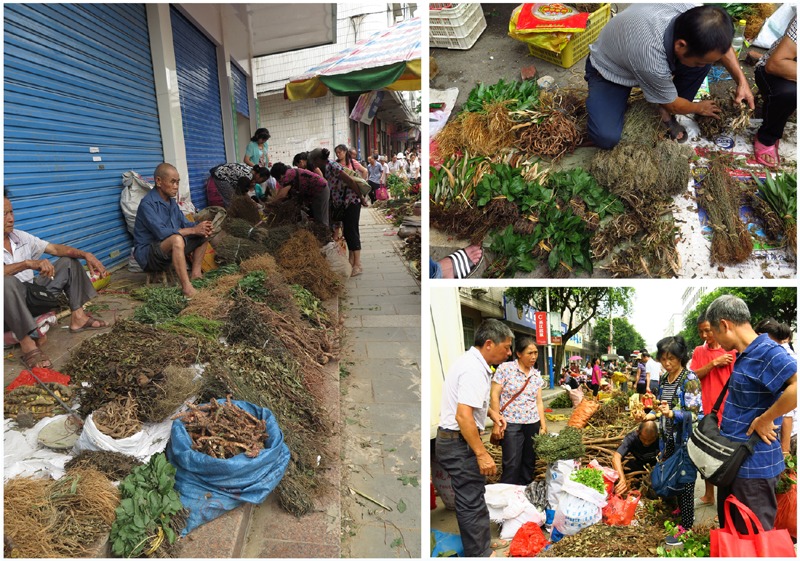
**Stall selling herbal and traditional remedies in China**.

Herbal medicines have played a significant role in the prevention and treatment of various diseases around the world since ancient times. At present, rapid industrialization and modernization exert profound effects on herbal medicine supply. Many herbal medicine products have found multiple ways onto store shelves and have become part of the self-medication of patients. The Internet has also become part of the marketplace, and some herbal products can be purchased without prescription. According to the World Health Organization [WHO] (2013), over three quarter of developing country populations rely on traditional medicine for their primary health care. The past decade has seen the strong growth of herbal product market worldwide (Friesen, 2013). The report from Global Industry Analysts Inc. estimated that the annual global sales of herbal products will reach up to 107 billion US dollar by the year 2017 (GIA, 2013). However, with the increase in the use of herbal medicines, reports on adverse reactions increase as well. In China, a country where traditional therapies and products are widely used in conjunction with conventional medicine, 9,854 cases of adverse drug reactions were reported in 2002 alone, with an increase of 4,000 between 1990 and 1999 (World Health Organization [WHO], 2004). In most of these cases, the cause of safety-related issues is the inaccurate identification of herbal materials. Plant misidentification may be due to similarities in appearance during bulk purchase or when harvested (Boullata and Nace, 2000; Liu et al., 2015). Confusing nomenclature or terms with common, transliterated, Latin, and scientific names could also lead to misidentification (Newmaster et al., 2013; Chen et al., 2014; Veldman et al., 2014; Wu et al., 2015). In addition to safety concerns, the quality of herbal products has also received increasing attention. Manufacturers are often tempted to increase their profit by incorrectly labeling their herbal products or adding low-priced ingredients of inferior quality to expensive ones; suspect or counterfeit herbal materials have also been found on sale (Ouarghidi et al., 2012; Xin et al., 2015; Mishra et al., 2016). For example, processed materials of the relatively rare herbaceous perennial *Panax ginseng* C. A. Mey are commonly adulterated with materials from the low-cost herb *Panax quinquefolius* (Chen et al., 2008). Contamination is also a repeated problem due to lack of quality control. Due to the cultural and historical influences, many unregistered and poorly regulated herbal products in some countries are sold freely on the stall with little or no restraint (**Figure 1**). Raw medicinal plant materials contaminated with each other may pose a potential health risk to the end users. Therefore, herbal medicine safety and quality problem needs to be solved immediately to safeguard customer health and ensure the quality and authenticity of herbal products in the drug supply chain.

To help address these issues, a vast array of detection and authentication methods and protocols have been developed for herbal medicine. Traditional methods include the use of morphology, microscopy, and chemistry (Franz et al., 2007; Li et al., 2010; Lau et al., 2012; Shen et al., 2014). These methods have been irreplaceable in the surveillance of herbal medicine from production and processing to marketing and surveillance since the emergence of pharmacognosy. However, these techniques have limitations. For example, although they are effective in the identification of fresh herbal plants, morphology approaches possess low effectiveness in the authentication of heavily processed herbal products (Li M. et al., 2011). Confusion is often caused by variations in chemical composition when chemistry-based methods are utilized (Wu et al., 2015). Even when the chemical composition of a product extract is known, the original plant may not be the specified one. In these cases, DNA-based methods are highly effective and can be applied to various herbal medicine materials. Furthermore, DNA is more informative than chemical composition and can be easily extracted in the presence of small traces of organic materials. Presently, methods that involve molecular biological techniques, such as sequence characterized amplified region markers (Adinolfi et al., 2007; Basha et al., 2009), amplified fragment length polymorphism (Percifield et al., 2008; Bandyopadhyay and Raychaudhuri, 2012), DNA microarray (Zhang et al., 2003; Liu et al., 2012), DNA barcoding (Chen et al., 2010; Mahadani and Ghosh, 2013; Pang et al., 2013; Xin et al., 2015), and high resolution melting (Osathanunkul et al., 2016) are available for herbal medicine identification. The major advantages of these methods are rapidity, high sensitivity, and specificity. However, these types of assays require expensive equipment and/or time costing, and the accuracy of their results dependent on trained experts. Recently, an isothermal molecular technology named loop-mediated isothermal amplification (LAMP) was introduced as an alternative to the use of polymerase chain reaction (PCR)-based methods in herbal medicine safety testing. Compared with PCR-based techniques, LAMP has a shorter reaction time and does not require specific equipment. Moreover, LAMP can be implemented with relatively impure sample materials as the template; time-consuming sample preparation prior to analysis is thus avoided. Although the application of LAMP in the identification and authentication of herbal products is still in the initial stage, LAMP has exhibited great potential in this field.

This article introduces the basic principle of LAMP functions and the important aspects in the development of LAMP analysis method, continue with a brief overview of the current role of LAMP in scientific studies, and then summarize the current information on the application of LAMP in herbal medicine identification. We also provide a practical standard operating procedure (SOP) for the utilization of the LAMP protocol in herbal medicine authentication, and finally discuss the prospects of using LAMP technology in the future developments of herbal medicine testing and the challenges associated with its application.

## Isothermal Amplification Technology

Nucleic acid amplification is an indispensable process in all living organisms. The process enables production of genetic copies to be passed onto the next generations for maintaining life. Nowadays, innovations in biotechnology are moving nucleic acid amplification from living organism to *in vitro* amplification techniques for molecular biology research. The nucleic acid amplification techniques can be categorized into two groups, non-isothermal nucleic acid amplification and isothermal nucleic acid amplification. One of the commonly used non-isothermal amplification techniques is PCR, which relies on thermocycling to replicate DNA and this characteristic has somewhat limited its application in low resource- and low skill setting places. Therefore, isothermal amplification techniques that represent a group of simple, portable and low-cost instruments nucleic acid amplification techniques for bioanalysis have been developed. We just list here the well known isothermal amplification techniques, such as hybridization chain reaction, helicase dependent amplification (HDA), Isothermal and chimeric primer-initiated amplification of nucleic acids, isothermal multiple displacement amplification, LAMP, nucleic acid sequence-based amplification (NASBA), rolling circle amplification, recombinase polymerase amplification (RPA), strand displacement amplification (SDA), signal mediated amplification of RNA technology, single primer isothermal amplification, transcription mediated amplification. Unlike PCR, isothermal amplification methods involve the amplification of DNA at a constant temperature and do not require thermocycling. The mechanisms of isothermal amplification technologies have been developed, and their capacities in on-site testing has been reviewed by many reviews (Deng and Gao, 2015; Li and Macdonald, 2015). These isothermal amplification assays offer high sensitivity and specificity when compared with the PCR-based methods (Craw and Balachandran, 2012; Yang et al., 2013), and this excellent characteristic has prompted the commercialization of some isothermal amplification technologies for rapid detection purposes by different companies, for example, LAMP (Eiken, Japan), NASBA (Coris Bioconcept, Belgium), RPA (Alere, USA and TwistDx, UK), SDA (Becton Dickinson, USA), and HDA (SAMBA, UK). Among them, LAMP is well-established and widely applied, with more than a thousand scientific articles concerning its application to many fields.

### LAMP Technology and Its Role in Scientific Researches

**FIGURE 2:**
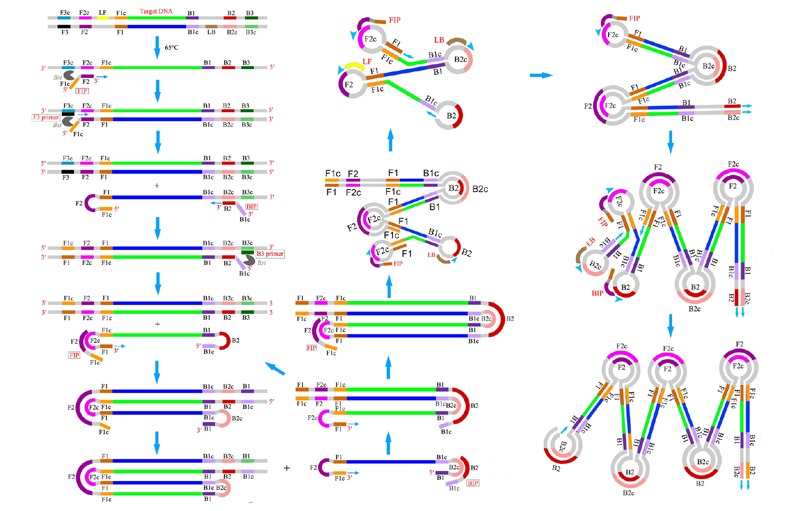
**Loop-mediated isothermal amplification scheme**.

LAMP is a revolutionary DNA amplification method based on strand displacement reaction and a stem-loop structure under isothermal condition (Notomi et al., 2000; Fukuta et al., 2004). It uses the *Bacillus stearothermophilus* DNA polymerase and a set of four to six specifically designed primers that hybridize to six or eight different parts of the target DNA sequence (**Figure 2**). Given the specific nature of the action of these primers, the amount of DNA produced in LAMP is considerably higher than that produced in PCR-based amplification (Mori and Notomi, 2009). After amplification, the amplified products are detected by the color change of the LAMP reaction solution from colorless to fluorescent green or via photometry for turbidity caused by an increasing quantity of magnesium pyrophosphate precipitate in the solution as a byproduct of amplification. This change can be easily observed with the naked eye, especially for large reaction volumes (Mori et al., 2013). The LAMP reaction does not require expensive equipment or special molecular techniques and is completed in a short time; consequently, this method is rapidly affecting the fields of molecular detection. At present, LAMP is one of the fastest amplification techniques that can be readily adapted for use in developing countries where there is a lack of infrastructure to support PCR facilities.

Since its emergence, LAMP has been utilized for the detection of various pathogens, including bacteria, viruses, and protozoans, that are relevant in human disease (Curtis et al., 2008; Njiru et al., 2008; Nagdev et al., 2011; Njiru, 2011; Bhadra et al., 2015), food contamination (Wang et al., 2008; Lee D. et al., 2009; Luo et al., 2012; Niessen et al., 2013; Huang et al., 2014; Yi et al., 2014), and plant pathology (Nie, 2005; Temple and Johnson, 2011; Duan et al., 2014; Kogovsek et al., 2015). Particularly over the past 10 years, the development of the number of publications involving the use of LAMP showed a multiplied nearly exponentially. In studies to detect any *Plasmodium* species in blood, or in faecal samples, they also have been verified the differentiation of the main *Plasmodium* species of human malaria: *P. falciparum, P. ovale, P. vivax*, and *P. malariae* (Han et al., 2007). In subsequent studies, LAMP has been further simplified to be in the form of a card test to diagnose malaria (Yamamura et al., 2009). Elsewhere, LAMP is used to distinguish between organisms differing by only a single nucleotide polymorphism to identify closely sub-genotypes (Ihiraa et al., 2008). LAMP analysis is also able to detect a single copy of bacteria in specimen and media samples. For example, a reverse transcription loop mediated isothermal amplification (RT-LAMP) combined with enzyme linked immunosorbent hybridization assay for *Mycobacterium tuberculosis* amplified from a single copy per reaction to detectable product in sputum samples, providing good agreement with real-time PCR applied to 150 clinical samples (Lee M. F. et al., 2009). Similar sensitivity accompanying specificity has been observed in HIV and malaria detection (Poon et al., 2006; Curtis et al., 2008). In addition to infection diagnosis, LAMP analysis plays an increasing role in food safety and quality testing. Bacterial pathogens are the main biological hazard in food safety, which can cause contamination due to poor food hygiene practices. In the case of a foodborne illness outbreak, the foodborne pathogens have to be identified in order to elucidate the source of contamination and to select an adequate treatment regime. Traditional culture-based methods require testing the pathogenic microorganisms by plating food samples to a series of selective or non-selective media. However, in some cases, culture-based methods may very well fail to detect a contamination properly due to the absence of viable cells of a pathogen. For these reasons, LAMP has been developed and is considered the rapid and sensitive method for the identification of food-related pathogenic microorganisms (Lee D. et al., 2009; Huang et al., 2014; Yi et al., 2014). In many cases commercial frauds are related to food adulteration problem, e.g., the fraudulent labeling of meat products. Several studies have focused on the identification of animal species using the LAMP assay (Lee et al., 2016; Ran et al., 2016). LAMP analysis is also suitable for genetically modified organism (GMO) screening and detection (Li et al., 2015). In this case, the LAMP targets can be commonly employed promoters (such as *P-35S*; Fukuta et al., 2004), specific genes (such as *cry1Ac*; Zhou et al., 2014), marker genes (such as *nptII*; Randhawa et al., 2013), and specific transgenic event (GM rice KMD1; Chen et al., 2012). Fukuta et al. (2004) introduced the LAMP method to detect the CaMV35S promoter in Roundup-Ready soybean, which is the first report of LAMP method applied to the GMO screening field. Lee D. et al. (2009) used LAMP in screening transgenic MS8 and RF3 oilseed rape (*Brassica napus*) and found that the detection limiting rate of this method was 0.01%. Zhang et al. (2013) developed a simple DNA extraction device combined with modified visual LAMP for the on-site detection of GMOs. In their study, high quality genomic DNA could be quickly obtained from plant tissues without using other laboratory instruments, LAMP can be performed without using a commercial thermal cycler, and visual inspection can be accomplished in tube without gel electrophoresis, this system will help greatly in meeting the demand for GMO in field testing. Zhou et al. (2014) published an optimized LAMP method to detect *cry1Ac* transgenic sugarcane, with the sensitivity of tenfold higher than that of conventional PCR. So far, a series of detection methods based on LAMP have been developed for GMO element detection, and they have been well summarized by Li et al. (2015), which therefore just to name a few here.

## Development and Application of LAMP in Herbal Medicine Identification

### Overview of LAMP Analysis Methods in Herbal Medicine Identification

Recent studies have demonstrated that LAMP analysis is an effective tool in herbal medicine identification (**Table 1**). In the following, we will critically discuss the development and application of LAMP analysis methods for herbal medicine identification.

**Table 1:** Overview of LAMP analysis methods applied in herbal medicine identification.

Aim	Target DNA region	Reference
Identification of *Curcuma longa* and *C. aromatica*	*trn*K chloroplast region	Sasaki and Nagumo, 2007
Detection of *Panax ginseng* and Ginseng	18S ribosomal RNA gene sequence	Sasaki et al., 2008
Identification and detection of *Cordyceps sinensis*	CS2 serine protease gene	Li K. et al., 2011
Authentication of *Catharanthus roseus*	Species-specific RAPD sequence	Chaudhary et al., 2012
Authentication of the herbal tea ingredient *Hedyotis diffusa*	ITS of the rRNA gene	Li et al., 2013
Screening toxic herb *Gelsemium elegans*	*trn*L-*trn*F chloroplast regions	Yu et al., 2014
Identification of the Herbal Tea Ingredient *Taraxacum formosanum*	ITS2 of the rRNA gene	Lai et al., 2015
Verification of the authenticity of *Aristolochia manshuriensis*	ITS2 of the rRNA gene	Wu et al., 2016
Authentication of the precious herb saffron	ITS2 of the rRNA gene	Zhao et al., 2016

Sasaki and Nagumo (2007) developed a LAMP-based method targeting *trn*K gene sequences to verify the identity of *Curcuma longa* and *C. aromatica*. They found that LAMP analysis is suitable for the identification of these two easily confused species (Sasaki and Nagumo, 2007). This is the first report on the utilization of the LAMP approach in rapid discrimination of herbal plants, and this study sparked a new research line on possible identification for herbal medicine products. In the following year, the same research group (Sasaki et al., 2008) published a similar LAMP analysis method for the detection of *Panax ginseng*, the botanical source of ginseng (Ginseng Radix), and to distinguish *P. ginseng* from *Panax japonicus*. By using a set of six allele-specific primers (two outer primers, two inner primers, and two loop primers), which are designed based on the partial 18S ribosomal RNA gene sequence, amplifications can be observed from approximately 30 min onward at DNA concentrations of 0.5 ng to 10.0 ng. Notably, although only three nucleotide differences exist in the 18S rRNA gene sequences of *P. ginseng* and *P. japonicus*, LAMP is highly sensitive to detect *P. ginseng* using these small nucleotide differences. Similar method was also devoted to the identification of traditional Chinese medicine *Cordyceps sinensis* (dong chong xia cao) and its adulterants *C. hawresii, C. ramosa, C. liangschanensis, C. militaris*, and *C. barnesi* (Li K. et al., 2011). In another study, LAMP can be combined with RAPD amplification to identify *Catharanthus roseus* (Chaudhary et al., 2012). These studies demonstrated that LAMP facilitates not only identification but also screening with high specificity.

In the past decade, DNA barcoding, a technique aiming at detecting species-specific differences in a short region of DNA, provides a powerful novel tool for herbal medicine identification. The short sequence of nucleotides (<1000 bp) used in this method can be easily obtained from an appropriate part of the chloroplast, mitochondrial, or nuclear genome and is commonly called “DNA barcode.” In 2010, Lou et al. (2010) developed a DNA barcoding database for medicinal plants^1^. This Medicinal plants DNA barcode database accepts all nuclear ITS and plastid DNA regions results for medicinal plants (Lou et al., 2010). More recently, Chen et al. (2014) established a universal, publically available DNA barcoding system based on the ITS2 and *psb*A–*trn*H barcodes for identifying medicinal plants. The database includes almost all crude herbal drugs listed in Chinese Pharmacopoeia, Japanese Pharmacopoeia, Korean Pharmacopoeia, Indian Pharmacopeia, United States Pharmacopeia, and European Pharmacopoeia. It also contains barcoding information for their adulterants, substitutes and closely related species. In order to make full use of these database and make LAMP method more universally and publically accessible, Li et al. (2013) presented a LAMP method targeting the DNA barcode region internal transcribed spacer (ITS) to verify its ability in the identity of *Hedyotis diffusa*. Lai et al. (2015) evaluated the effectiveness of ITS2 DNA barcode in distinguishing *Taraxacum formosanum* from other adulterant plants using LAMP method. These studies suggested that DNA barcodes could be used to design species-specific primer for LAMP to distinguish medicinal species from their non-medicinal adulterants. Very recently, Zhao et al. (2016) developed a similar approach for the identification of *Crocus sativus* through LAMP analysis. The authors constructed multiple alignments of the ITS2 sequences between *C*. *sativus* and its adulterants and found this sequence is suitable to design *C. sativus* LAMP-specific primers. In particular, 10 fg of genomic DNA is identified as the limit for template accuracy of LAMP in *C*. *sativus*, indicating that LAMP is more sensitive than normal PCR, which typically requires a minimum template amount of 10 ng. The authors provided a practical SOP for utilizing the LAMP protocol in herbal authentication. In the near future, people can use the LAMP method combined with DNA barcodes to test their herbal materials according to this SOP.

In markets, herbal products are commonly sold without packaging or labels, leading to a high risk of acquiring counterfeit, substituted, and/or adulterated products. Traditional herbal medicines that are adulterated and/or contaminated with toxic plant materials have caused a number of major epidemic events. For example, in the early 1990s, an epidemic of urothelial carcinomas related to AAs occurred in Belgium. A number of women were prescribed the Chinese herb Fangji (*Stephania tetrandra*) for weight loss, but the herb was incorrectly substituted with AAs containing Guangfangji (*Aristolochia fangchi*). Subsequently, the women developed renal interstitial fibrosis and upper tract urothelial carcinomas-aristolochic acid nephropathy (Vanherweghem et al., 1993). In another case, a woman prescribed herbal preparations for the treatment of hypercholesterolemia suffered from hepatitis after using preparations containing germander (*Teucrium chamaedrys*). The main reason for this occurrence is the incorrect substitution of adulterants, such as germander (*Teucrium chamaedrys*) instead of skullcap (*Scutellaria*) (Lord et al., 1999). Any case regarding herbal medicine safety, especially when reported by the media, significantly influences public opinion. Thus, the demand for the improvement of quality control for herbal medicine continues to increase.

The screening of toxic adulterants and admixtures in herbal products is one of the more important applications of LAMP analysis. Yu et al. (2014) evaluated LAMP method targeting the plant DNA barcoding regions *trn*L–*trn*F to verify the identity of the toxic herb *Gelsemium elegans*. The aim was to distinguish *Lonicera japonica* from *G*. *elegans*, which could be mislabeled as an adulterant in commercial *L*. *japonica* products due to their similar appearance. The research began by sequencing standard regions of the plastid DNA (*trn*L-F and *trh*H-*psb*A) of these two species. Multiple alignment results found that *trn*L–*trn*F amplicons is suitable to design species-specific primers for the identification of *G*. *elegans*. Based on their study, the authors developed a LAMP kit that can be adapted for this toxic adulterant screening. In another study, Wu et al. (2016) developed a real time LAMP assay for the detection of *Aristolochia manshuriensis* which has been reported to be the most common toxic adulterant in *Akebiae Caulis* herbal products. The authors also highlighted the limit detection of LAMP. In their study, the identity of *Aristolochia manshuriensis* in processed commercial herbal products can be verified as low as 1%.

These case studies and technical advancements clearly indicate that LAMP identification can be applied to a wide range of herbal materials from the field, commercial trade, hospital pharmacies, etc. If the result indicates that the species is different from the anticipated species, then the former may be an accidental or intentional substitute that may or may not be harmful. By determining that an item is not the correct species, the use of that sample (and presumably its entire batch) can be avoided, and a potential adverse reaction could be prevented. Similarly, LAMP technology may also assist the herbal pharmacovigilance sector in resolving the causes of adverse reactions. Such applications of this technology can help improve the safety of herbal medicines in general, especially when combined with authentication via morphological, chemical, and/or other molecular methods.

### Developing a Practical SOP for Rapid Detection of Herbal Medicines Using LAMP

**FIGURE 3:**
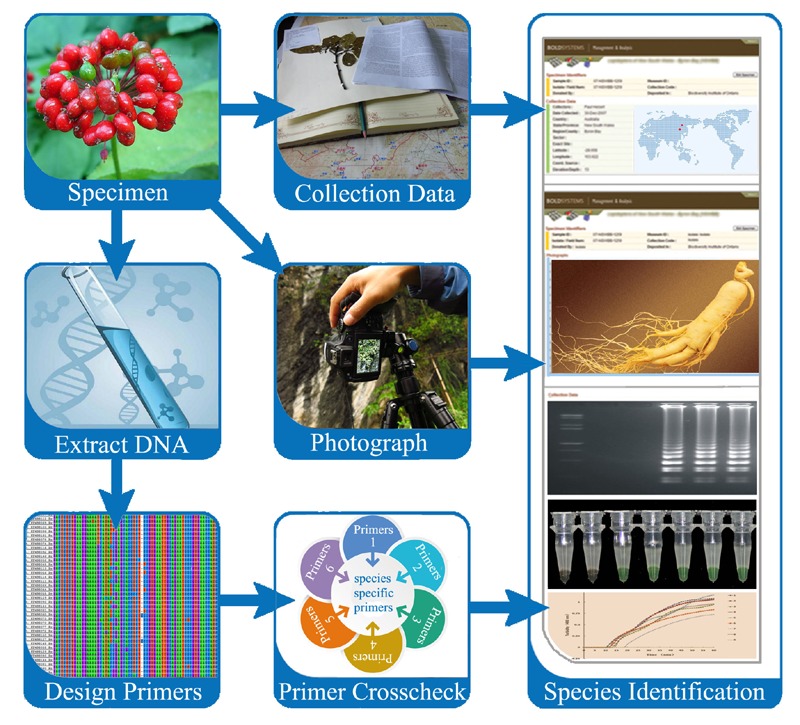
**A proposed SOP for herb authentication utilizing a LAMP protocol**.

The LAMP method has been well-established and widely applied in many fields, such as the detection of human diseases and plant pathology, testing of bacterial pathogens and fungal contaminants in foods, and screening GMOs. The need for an immediate identification method is urgent in the herbal medicine market. To address this, we refer to related literatures and propose a practical SOP for herbal medicine identification utilizing the LAMP protocol (**Figure 3**). Establishing a SOP for herbal medicine identification involves sample collection, verification of voucher herbarium specimens, DNA extraction, candidate DNA region sequencing, species-specific LAMP primer design, LAMP amplification, and finally, species testing and product screening.

#### Sample Collection and Morphological Verification

To design reliable species-specific LAMP primers, assurance about the identity of the source plants is vital. At least three duplicate collections per species should be used; each collection (uniquely numbered) must include a small plant sample for DNA extraction and voucher herbarium specimens of the entire plant (ideally flowering or fruiting) and must be sourced from the same plant or population of plants. The collections should be accompanied by photos and detailed field notes describing any identifying characteristics not evident from the herbarium specimens. The samples must be in good condition (e.g., devoid of contamination). A detailed guide to making herbarium specimens can be found in *The Herbarium Handbook* (Bridson and Forman, 1992).

#### DNA Extraction

High-quality genomic DNA is an essential prerequisite to ensure normal amplification in PCR and accurate identification through LAMP analysis. DNA extraction from the herbal material must therefore be performed carefully and quickly using a good sterile technique to avoid DNA degradation and contamination. Referring to related literatures and our recent studies (Kool et al., 2012; Chen et al., 2013; Xiang et al., 2013; Xin et al., 2013; de Boer et al., 2014), high-quality DNA samples can be obtained by using the modified CTAB method (Sun et al., 2016). Herein, we summarize the key points to consider in the DNA extraction of herb materials. Herbal materials are usually harvested long before they are used; hence, the surface of herbal materials should be cleaned with 75% alcohol prior to being ground to fine powder in liquid nitrogen for DNA extraction. The high levels of polysaccharides and polyphenols in some herbal materials must be removed with polyvinylpyrrolidone (PVP) and β-mercaptoethanol during the early stages of DNA extraction. In most cases, medicinal plant products are commonly sold in processed or modified forms, such as dried materials, tablets, or powders, making their DNA severely degraded. Thus, for DNA extraction, the dosage of plant starting material, PVP, and β-mercaptoethanol must be increased accordingly.

#### DNA Region Selection

Selecting an appropriate DNA region is essential for successful analysis. The GC content, Tm value, secondary structure, and primer location in the region are the limiting factors for primer design. As a general principle, the DNA fragment must be conserved, and the sequence length should be sufficiently long to contain all of the regions adapted to design LAMP primers (Zhao et al., 2016). For this reason, not all common fragments result in highly efficient LAMP primers. Therefore, we recommend designing several groups of LAMP primers based on different fragments.

#### Species-Specific LAMP Primer Design

LAMP reactions result in very high specificity because of the use of specifically designed primers. LAMP primer design is similar to that of PCR, but significant differences exist between the two approaches. LAMP primers entail several critical factors, such as primer and amplicon length, melting temperature (Tm), GC content, secondary structure, stability at the end of each primer, and distance between primers (Notomi et al., 2000; Zhao et al., 2016). The primer design conditions for a normal sequence (45% < GC < 60%) are inputted as a default setting. If the target sequences are AT rich (GC content < 45%), then the LAMP primers (F3 and B3) should be designed with Tm > 55°C, length (18–25 nt), and GC content < 45%. If the target sequences are GC rich (GC content > 60%), then the LAMP primers (F3 and B3) should be designed with Tm < 68°C, length (15–22 nt), and GC content > 60%. After the four regular LAMP primers (FIP, BIP, F3, and B3) have been determined, the loop primers can be designed based on the primer information file of the regular primer set. Although the loop primers can dramatically reduce the amplification time, they are not the essential requirement for LAMP. Designing primers to produce a resultant amplicon size of <300 bp enables good sensitivity and reaction speeds. In principle, amplicon size that exceeds 500 bp can be obtained, but these long amplicons can result in poor sensitivity and long reaction times. For the best amplification, the end of the primers serves as the starting point of the DNA synthesis and must thus have a certain degree of stability. The 3′ ends of F2/B2 and F3/B3 and the 5′ end of F1c/B1c are designed in such a manner that the free energy is -4 kcal/mol or less. The 5′ end of F1c after amplification corresponds to the 3′ end of F1, so stability is important. The success of LAMP also depends on the distance between primers. The primers are designed in such a manner that the distance from the end of F2 to the end of B2 is between 120 and 160 bases. The primers are also designed such that the distance from the 5′ end of F2 to the 5′ end of F1 (the portion that forms the loop) is between 40 and 60 bases. The primers are also designed such that the distance between F2 and F3 is between 0 to 60 bases. LAMP primer sets can be designed with Primer Explorer, which is specifically for LAMP^2^. This software has special characteristics to design and shortlist four superior LAMP primer sets, followed by checking the GC content, stability at the end of each primer, and other key parameters. For herbal medicine identification, on the basis of the interspecies and intraspecies sequence variations of the selection DNA region, we summarize the key points as follows. First, the length of F1c/B1c is 18–23 bp, whereas the lengths of F2/B2 and F3/B3 are 18–22 bp. Second, the melting temperature for F1c/B1c is 60–65°C, whereas the Tm for F2/B2 and F3/B3 is 58–61°C. The melting temperature should be adjusted to the appropriate range according to the characteristic of the amplified products. Third, the distance between F2 and B2 is 50–150 bp, whereas the distance between F1c and B1c is 0–50 bp. The concentration of each primer is also important to obtain a good result. Generally, the concentrations (inner primers > loop primers > outer primers) are design to optimize the reaction system.

#### LAMP Amplification and Herbal Product Identification

The reaction conditions and components used for LAMP amplification can be found in a previous study (Li et al., 2013; Wu et al., 2016; Zhao et al., 2016). Briefly, genomic DNA from the original plant of the herbal material and its adulterant are used as templates. LAMP amplification is performed with different groups of primers to crosscheck their species-specific characteristics. The best group of primers that are both stable and efficient are then used to screen commercial herbal products. Species identification is accomplished by analyzing the turbidity curve in combination with visual color change instead of using only the DNA electrophoresis test. Given the high sensitivity of the LAMP test, the occurrence of false positive results is likely in the current experimental setup. To prevent such results, the use of low-melting point paraffin wax to reduce the probability of contamination is recommended.

## Future Opportunities and Challenges of LAMP in Herbal Medicine Identification

Since herbal medicines remain largely unregulated, consumers worldwide need to be informed and given the tools to access appropriate, safe and effective treatment. To help address this issue, many emerging technologies (including chemical and molecular) have been utilized in herbal medicine identification. However, their application is still limited partly due to the infrastructures require in the laboratory and skills require well-trained experts. Most developing countries still focus on the use of traditional methods (e.g., morphology and microscopy) to identify herbal medicines. With the recent invention and advancement of isothermal technologies, molecular isothermal amplification has become reality. Among these novel isothermal technologies, LAMP holds particular potential for use in herbal medicine identification, this technology has salient advantages over most PCR-based amplification tests and has dramatically changed our view on application of molecular technologies, which were previously regarded as difficult to apply in the poorly resourced laboratories. LAMP bridges the technology gap between traditional identification methods and PCR-based methods. At present, LAMP has been well established and widely applied, with more than thousand scientific articles concerning its application to clinical diagnosis and food testing. It is known that LAMP reagents have been commercialized in several ready to use kits, identification of herbal medicine through the use of LAMP assay kits that combine simplicity, rapidity, and high efficiency is an effective approach, it simplifies the complicated technicality of molecular detection of herbal products and can significantly contribute to the control of adulteration in the drug supply chain, especially in settings where lack of infrastructure to support PCR facilities. Nevertheless, this technology is still rarely adopted for herbal medicine identification in developing countries where they are highly needed. Implementation of LAMP technology in developing countries may help identify the impediments that prevent the adoption of new molecular technologies in low-resource and low-skill settings. Therefore, in the future, increased adoption of this technology in the detection of herbal products is expected.

Robustness of the process, testing devices, and extensive and accessible primer resources are the most relevant properties for the routine of LAMP in herbal medicine identification. Despite it proven effectiveness, the application of LAMP method in the authentication of herbal products is still in the initial stage, and its use in the context of herbal product traceability remains limited due to the lack of LAMP primers. Additional extensive studies are required to exploit and establish a primers database for LAMP as a reliable detection tool in herbal medicine identification. In addition, international agencies or institutions that are responsible for quality control of raw medicine materials or processed herbal products can cooperate by sharing data, hence creating population reference databases. Many established techniques suffered from a similar limitation in the beginning but have developed into robust, universal tools (e.g., DNA barcoding). In fact, while some groups of herbal medicine species are well represented, a great deal of work is required to provide an available source of reference LAMP primer data for groups which have been poorly investigated. Therefore, continual updating will help to ensure the integrity of the LAMP primer database and broaden its application. Besides, it is very important to note that the high specificity and sensitivity of LAMP can sometimes lead to false-positive amplification due to cross contamination, caused especially by aerosol in the assay process (Tomita et al., 2008). Previous studies revealed that detection involve process of opening the reaction tube after reaction, are easy to produce aerosols (Wang et al., 2009; Zhou et al., 2014). Therefore, it is necessary to pay a great attention for the treatment of biological samples. In order to avoid cross contamination, the closure of reaction tube caps, dual filter tips, and disposal in plastic bags is recommended whenever possible (Wang et al., 2009). Furthermore, 75% ethyl alcohol disinfection, ultra-violet sterilization and a clean bench may be necessary (Zhou et al., 2014). Another critical challenge with LAMP assay development is its difficulty in quantification, even though real-time turbidimetry allows the quantification of the template DNA in the reaction with the possibility of analyzing minute quantities of nucleic acids (Abdul-Ghani et al., 2012). Often, researchers determine the amount of a given sample by establishing linear regression analysis according to the change in real time turbidity. In all studies a correlation was found between the turbidity level and the amount of target object in the mixture. However, in all cases the linear regression analysis was just established in spite of the fact that an optical evaluation of the data points hardly justifies. So far, some critical discussion of key issues, e.g., the influences of sample contamination and matrix effects on the sensitivity, specificity and accuracy of the methods, is missing. In our opinion, LAMP analysis method has to be evaluated in more detail in order to demonstrate its applicability for the accurate quantification.

## Conclusion

The advent of LAMP to identify plant species in herbal medicine appears to be promising but remains to be fully exploited. Insofar, LAMP-based methods will not replace classical PCR in the near future but may provide a supplementary tool in applications in which presence or absence of the target DNA sequence in the herbal medicine product needs to be detected to facilitate decisions about further analysis.

## Author Contributions

J-jL and CX wrote the manuscript. YL helped to collect reference articles. J-sL and X-wZ helped with language editing support and provided helpful comments on the article.

## Conflict of Interest Statement

The authors declare that the research was conducted in the absence of any commercial or financial relationships that could be construed as a potential conflict of interest.

## Footnotes

1 http://www.cuhk.edu.hk/icm/mmdbd.htm

2 http://primerexplorer.jp/elamp4.0.0/index.html
